# Baseline malaria vector transmission dynamics in communities in Ahafo mining area in Ghana

**DOI:** 10.1186/s12936-015-0667-6

**Published:** 2015-04-07

**Authors:** Dominic B Dery, Kwaku P Asante, Charles Zandoh, Lawrence G Febir, Charles Brown, George Adjei, Yaw Antwi-Dadzie, Emmanuel Mahama, Kofi Tchum, David Dosoo, Seeba Amenga-Etego, Robert Adda, Christine Mensah, Kwabena B Owusu-Sekyere, Chris Anderson, Gary Krieger, Seth Owusu-Agyei

**Affiliations:** Kintampo Health Research Centre, Ghana Health Service, Ministry of Health, P.O. Box 200, Kintampo, Ghana; College of Health Sciences, University of Ghana, Legon, Ghana; Newmont Ghana Gold Limited, C825/26 Lagos Avenue, East Legon, Accra, Ghana; HealthLink Consulting, P.O. Box AN 6811, Accra-North, Ghana; Newfields, 730 17th Street, Suite 925, Denver, CO 80202 USA

**Keywords:** Malaria transmission, Ahafo, *Anopheles gambiae*, *Anopheles funestus*

## Abstract

**Background:**

Malaria vector dynamics are relevant prior to commencement of mining activities. A baseline entomology survey was conducted in Asutifi and Tano (referred to as Ahafo) in the Brong-Ahafo geo-political region of Ghana during preparatory stages for mining by Newmont Ghana Gold Limited.

**Methods:**

Between November 2006 and August 2007, eight Centre for Disease Control light traps were set daily (Monday-Friday) to collect mosquitoes. Traps were hanged in rooms that were selected from a pool of 1,100 randomly selected houses. Types of materials used in construction of houses were recorded and mosquito prevention measures were assessed from occupants.

**Results:**

A total of 5,393 mosquitoes were caught that comprised *Anopheles gambiae* (64.8%), *Anopheles funestus* (4.2%), as well as Culicines, comprising of *Culex* (30.4%) and *Aedes* species (0.6%). The entomological inoculation rate in Asutifi (279 infective bites/person/month) and Tano (487 infective bites/person/month) demonstrate relatively high malaria transmission in Ahafo. The presence or absence of *Anopheles* vectors in rooms was influenced by the type of roofing material (OR 2.33, 95%CI: 1.29-4.22, p = 0.01) as well as the presence of eaves gaps (OR 1.80, 95%CI: 1.37-2.37, p < 0.01). It was also associated with bed net availability in the room (OR 1.39, 95%CI: 1.08-1.80, p = 0.01). Over 80% of the houses were roofed with corrugated zinc sheets. Over 60% of the houses in Ahafo had no eaves gaps to give access to mosquito entry and exit into rooms and mosquito bed net coverage was over 50%. Other measures used in preventing mosquito bites included; coil (22.1%), insecticide spray (9.4%), repellent cream (4.0%) and smoky fires (1.1%), contributed minimally to individual mosquito preventive measures in impact areas. Similarly, levels of protection; coil (16.9%), insecticide spray (2.8%) and repellent cream (0.3%) for the non-impact areas, depict low individual prevention measures.

**Conclusions:**

The survey identified areas where intensified vector control activities would be beneficial. It also demonstrates that transmission in Asutifi and Tano is high even before the commencement of mining operations. This study serves as baseline information to assess impact of mining activities in relation to future vector control interventions.

## Background

Knowledge of vector bionomics is essential in understanding the epidemiology and implementing control of malaria [1]. Today, malaria can be prevented, diagnosed and treated with a combination of well-established strategies. Vector control is one of the key control tools and involves the use of insecticide-treated mosquito nets (ITN), indoor residual spraying and larval control activities [2]. Malaria vector control is an important strategy recognized in the global policy frameworks [3,4] of which Ghana is no exception.

Ghana is endowed with significant mineral deposits [5]. These deposits have attracted both large international mining companies and small-scale artisanal miners. Mining activities can lead to land degradation, changes in landscape, deforestation and changes in human behaviour [6]. More importantly, mining activities may lead to a change in the abundance and distribution of malaria vectors [7,8]. Mining activities may also lead to enhanced household income and improved housing construction, which can impact on malaria transmission.

The attraction of mosquitoes into a house or room is dependent on various factors including construction materials and room characteristics [9,10]. Studies in Sri Lanka and Kenya [11,12] found that the risk of getting malaria was greater for inhabitants occupying the poorest type of houses which was characterized by incomplete construction with thatched roofs and walls made of mud or *cadjan* (woven coconut palm leaves). This was compared with better-constructed houses made of complete brick and plastered walls and tiled roofs. The Kenyan study specifically found 84% (OR 0.16, 95% CI 0.07-0.39, P < 0.0001) and 87% (OR 0.13, 95% CI 0.03-0.5, P = 0.0004) reduction in the odds of finding *Anopheles gambiae s.l.* and *Anopheles funestus* in better constructed houses compared with poorly constructed houses [13]. Thus, malaria transmission may be affected by improvement in housing construction as a result of enhanced income generation in mining areas.

Malaria assessment, including vectorial capacity determination, are important prior to introduction of mining activities as this will inform monitoring of vector dynamics over time so as to plan effective control interventions. Prior to the start of active mining in the Ahafo area of Ghana, Newmont Gold Ghana Ltd (NGGL) commissioned a variety of environmental, social and health baseline studies. The present study was commissioned by NGGL in order to determine basic malaria entomological indices including transmission dynamics in the mining area.

## Methods

### Study areas and communities

**Figure 1:**
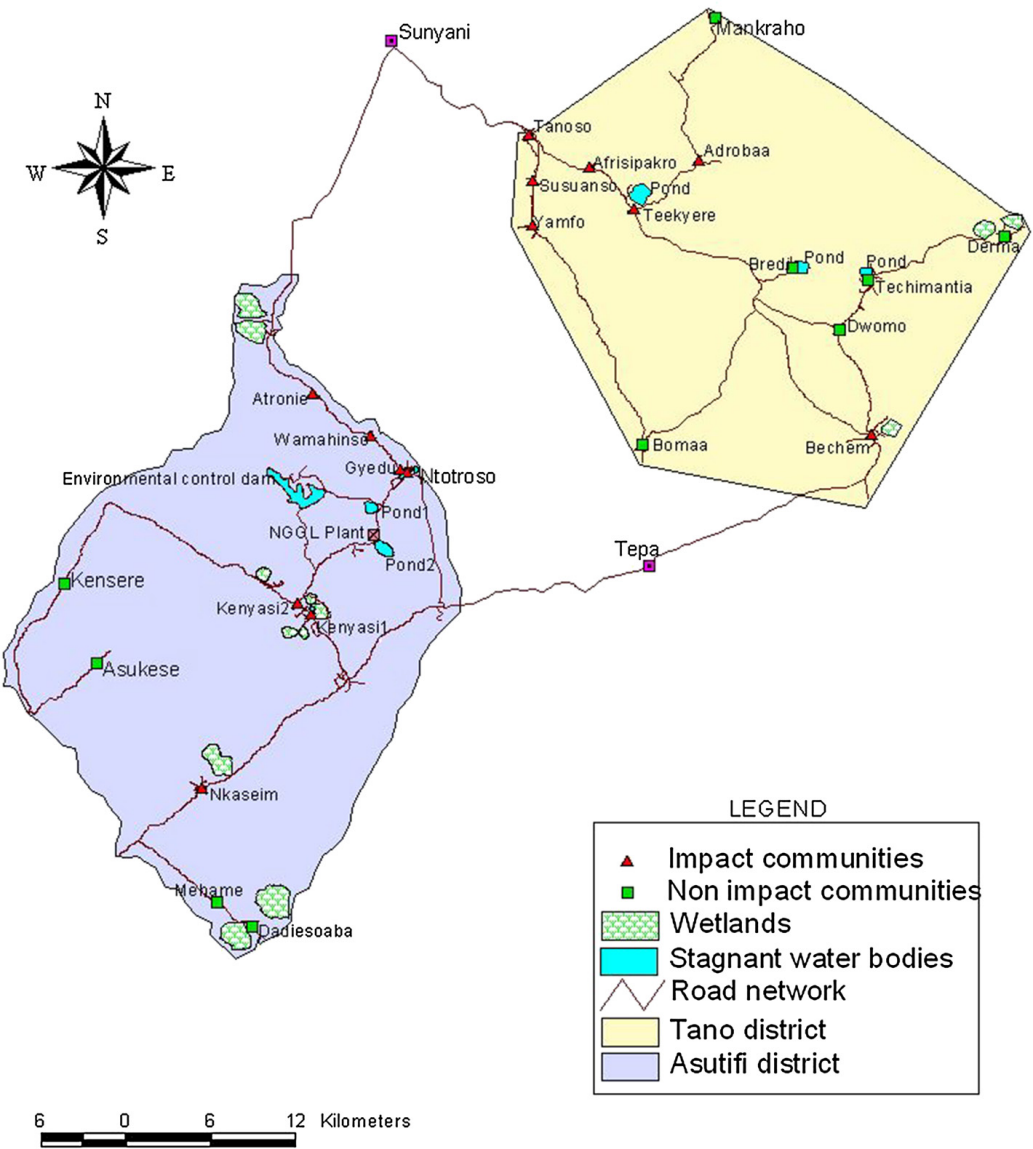
**Map of Asutifi and Tano area in Ghana (Nov 2006-Aug 2007).**

**Figure 2:**
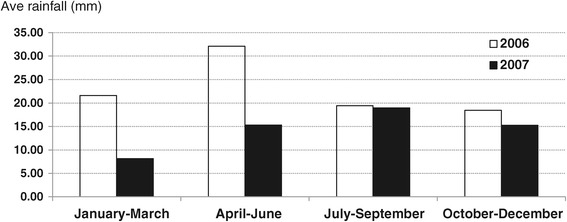
**Bi-monthly rainfall pattern in Asutifi and Tano area in Ghana (2006–2007).**

The study was conducted in two areas in the Brong Ahafo Region of Ghana; Asutifi and Tano (Figure 1). Asutifi lies between latitudes 6°40′ and 7°15′ North and Longitudes 2°15′ and 2°45′ West. Tano lies between latitudes 7°00′N and 7°25′ N and between longitudes 1°45 W and 2°30 W. Asutifi had a population of approximately 97,977 and land surface area of 1,500 square kilometres. Tano had a total population of approximately 155,100 at the time of survey. The study area falls within the wet semi-equatorial forest zone where mean annual rainfall is about 1200 mm per annum. The quarterly rainfall pattern in the area (Figure 2) depicts rainfall throughout the year and reflects two raining seasons in the area. The major economic activities in the study areas besides gold mining are farming of cash crops, such as cocoa, palm oil, yam and plantain.

Studied communities were grouped into “impact” and “non-impact” communities based on the assumption that mining operations could have an impact on the health and socio-economic status of the population. “Impact” area means communities either in the Asutifi or Tano area whose health and/or socio-economic indices are likely to be directly affected by mining activities being undertaken and located within 25 Km radius from the mine sites. The “non-impact” area represents communities in either the Asutifi or Tano areas that are not likely to be affected directly by the mining activities and located outside the 25 kilometres radius from the mines site.

The area has a health system conforming to the basic primary care model established by the Ghana Health Service. Communities are served by a local health clinic that manages basic health problems. All health facilities in the study area render both clinical and public health services. Malaria is the leading reported cause of outpatient attendance in all health facilities in the study area.

### Study design and methods

Houses were randomly selected from among those used in a district-wide household morbidity survey [14] and mosquito traps were set using CDC light traps. All the houses in the morbidity survey that had children less than five years of age formed the sampling frame from which rooms were selected for the mosquito light trappings.

A total of 1,100 houses were randomly selected from enumerated houses in the Asutifi and Tano areas and used for the weekly trappings. Eight CDC light traps were set daily; four were set simultaneously in Asutifi and Tano respectively, starting from November 2006 and ending in August 2007. Traps were set at the foot-end of the person(s) sleeping in the selected room and hanged approximately 1.6 metres above the floor. Untreated mosquito nets were provided to household members whose rooms were used on the night of trap setting as an ethical requirement.

The characteristics were recorded for each house where a trap was set; these included the types of materials used for roof construction, walls and floor; presence of eaves gaps, number of windows and doors. Malaria vector preventive measures were assessed one week prior to the date of survey on occupants in each room that received a light trap.

Mosquitoes caught were chloroformed and morphologically identified using Anopheline morphological identification keys [15]. They were then stored in 1.5 ml eppendorf tubes and transported on weekly basis to the Kintampo Health Research Centre laboratories for enzyme-linked immunosorbent assay (ELISA) [16]. An average cut-off point of 0.2 nm absorbance wavelength was considered positive. Positive samples were re-tested for confirmation.

### Data management

All data collected in the field or the laboratory were logged for traceability, and then batched for double data entry and processing using Microsoft® Access.

### Statistical analysis

The entomological inoculation rate (EIR) was calculated as the product of the proportion of *Anopheles* positive by ELISA also termed as sporozoite rate (SR) and the man biting rate, which was estimated as the geometric mean of *Anopheles* caught by CDC light traps per night. The monthly EIR (EIRm) was calculated as the product of EIR for a night (EIRn) and estimated number of days in a month (30). The presence or absence of Anopheline species in houses in relation to housing construction and location was modeled using logistic regression controlling for the main confounding variables.

### Ethics

Approval for the study was obtained from the Kintampo Institutional Ethics Committee (Federal Wide Assurance number: 00011103). Participants were consented the night before setting a light trap. Untreated mosquito nets were provided to occupants of rooms on the night of setting trap to ensure they were protected while ensuring that mosquitoes were not repelled. The Kintampo Health Research Centre ethics committee approved this method.

## Results

### Mosquito vector abundance and EIRs in Asutifi and Tano

A total of 942 CDC light trap-nights were set within the period (November 2006 – August 2007). A total of 5,393 mosquitoes were collected which comprised *An. gambiae* (64.8%), *An. funestus* (4.2%), *Culex* species (30.4%) and *Aedes* species (0.6%). Mosquito abundance was generally higher in the impact areas of Asutifi compared with the non-impact areas (Table 1). Ntotroso recorded the highest number of mosquitoes caught in the impact area and Mehame recorded the highest mosquitoes caught in non-impact area. *An. gambiae* and *An. funestus* vector abundance in the two areas were significantly different. *Anopheles gambiae* was much higher (ten-fold) in impact areas compared to *An. funestus*. However, in non-impact areas abundance of both species were nearly equal.

**Table 1:** **Mosquito abundance and EIRs in Asutifi area in Ghana (Nov 2006 – Aug 2007)**

**Location**	**VILLAGE**	***An. gambiae n*** **(%)**	***An. funestus n*** **(%)**	***Culex sp.*** **n (%)**	***Aedes sp.*** **n (%)**	**SR-Ag**	**SR-Af**	**SR**	**MBR**	**EIRn**	**EIRm**
**IMPACT**	ATRONIE	41 (4.7)	1 (0.5)	47 (4.6)	7 (50.0)	0.1	0.0	0.1	6.0	0.6	18.0
	GYEDU	48 (5.5)	13 (6.8)	81 (7.9)	0 (0.0)	0.3	0.0	0.3	17.0	4.3	127.5
	KENYASI NO.2	44 (5.1)	2 (1.1)	108 (10.6)	0 (0.0)	0.3	0.0	0.3	2.0	0.5	15.0
	NKASEIM	67 (7.7)	42 (22.1)	95 (9.3)	1 (7.1)	0.1	0.1	0.2	7.0	1.2	35.7
	NTOTROSO	309 (35.7)	14 (7.4)	175 (17.2)	6 (42.9)	0.1	0.0	0.1	8.0	0.3	9.6
	WAMAHINSO	38 (4.4)	1 (0.5)	48 (4.7)	0 (0.0)	0.1	0.0	0.1	6.0	0.3	9.0
	KENYASI NO.1	201 (23.2)	16 (8.4)	125 (12.3)	0 (0.0)	0.1	0.1	0.2	8.0	1.0	31.2
**NON-IMPACT**	ASUKESE	34 (3.9)	9 (4.7)	37 (3.6)	0 (0.0)	0.2	0.0	0.2	5.0	0.8	24.0
	DADIESOABA	50 (5.8)	25 (13.2)	102 (10.0)	0 (0.0)	0.1	0.0	0.1	17.0	1.4	40.8
	KENSERE	11 (1.3)	34 (17.9)	48 (4.7)	0 (0.0)	0.2	0.0	0.0	5.0	0.2	6.0
	MEHAME	22 (2.5)	33 (17.4)	153 (15.0)	0 (0.0)	0.1	0.0	0.1	7.0	0.6	16.8
	**Total**	**865**	**190**	**1,019**	**14**	**1.4**	**0.2**	**1.4**	**6.9**	**9.3**	**278.6**
	**TOTAL** ^**a**^	**2,088** ^**a**^									

**SR**-Ag; Sporozoite Rate for *An. gambiae*, **MBR**; Man Biting Rate.

**SR**-Af; Sporozoite Rate for *An. funestus*, **EIRn**; Entomological Inoculation Rate/night.

**EIRm**; Entomological Inoculation Rate/month, **SR**; Sporozoite Rate (Ag + Af).

**Superscript (** ^**a**^ **) -** Total mosquitoes caught in Asutifi **n;** number caught.

Relatively higher numbers of mosquitoes were caught in non-impact areas than impact areas in Tano (Table 2). Afisipakrom recorded the highest number of mosquitoes caught in impact area and Bredi recorded the highest number of mosquitoes in non-impact area. Most *Anopheles* vectors caught were *An. gambiae* with nearly negligible numbers of *An. funestus* vectors.

**Table 2:** **Mosquito abundance and EIRs in Tano area in Ghana (Nov 2006 – Aug 2007)**

**Location**	**VILLAGE**	***An. gambiae*** **n (%)**	***An. funestus*** **n (%)**	***Culex sp.*** **n (** ***%*** **)**	***Aedes sp.*** **n (%)**	**SR-Ag**	**SR-Af**	**SR**	**MBR**	**EIRn**	**EIRm**
**IMPACT**	ADROBAA	94 (3.6)	1 (2.7)	20 (3.2)	0 (0.0)	0.1	0.0	0.1	11.0	0.6	16.5
	AFISIPAKROM	448 (17.0)	2 (5.4)	23 (3.7)	0 (0.0)	0.1	0.0	0.1	36.0	1.8	54.0
	BECHEM	93 (3.5)	3 (8.1)	182 (29.4)	0 (0.0)	0.1	0.0	0.1	6.0	0.8	23.4
	SUSUANSO	17 (0.6)	1 (2.7)	24 (3.9)	0 (0.0)	0.1	0.0	0.1	4.0	0.2	7.2
	TEEKYEERE	61 (2.3)	1 (2.7)	10 (1.6)	0 (0.0)	0.2	0.0	0.2	8.0	1.2	36.0
	TANOSO	27 (1.0)	1 (2.7)	31 (5.0)	1 (50.9)	0.2	0.0	0.2	4.0	0.7	21.6
	YAMFO	18 (0.7)	0 (0.0)	45 (7.3)	1 (50.9)	0.3	0.0	0.3	3.0	0.8	25.2
**NON-IMPACT**	BOMAA	69 (2.6)	5 (13.5)	43 (6.9)	8 (47.1)	0.3	0.0	0.3	5.0	1.5	45.0
	BREDI	1321 (50.2)	0 (0.0)	16 (2.6)	0 (0.0)	0.0	0.0	0.0	274.0	5.5	164.4
	DERMA	147 (5.6)	1 (2.7)	104 (16.8)	1 (5.9)	0.1	0.0	0.1	11.0	1.1	33.0
	DWOMO	217 (8.2)	13 (35.1)	39 (6.3)	1 (5.9)	0.0	0.0	0.0	31.0	0.9	27.9
	MANKRAHO	64 (2.4)	8 (21.6)	64 (10.3)	4 (23.5)	0.2	0.0	0.2	6.0	1.0	28.8
	TECHIMANTIA	56 (2.1)	1 (2.7)	18 (2.9)	1 (5.9)	0.1	0.0	0.1	4.0	0.4	13.2
	**Total**	**2,632**	**37**	**619**	**17**	**1.7**	**0**	**1.6**			**486.8**
	**TOTAL** ^**b**^	**3,305** ^**b**^									
		**3,497 (64.8)**	**227 (4.2)**	**1,638 (30.4)**	**31 (0.6)**						
	**TOTAL (Tano + Asutifi)**	**5,393**									

**SR**-Ag; Sporozoite Rate for *An. gambiae*, **MBR**; Man Biting Rate.

**SR**-Af; Sporozoite Rate for *An. funestus*, **EIRn**; Entomological Inoculation Rate/night.

**EIRm**; Entomological Inoculation Rate/month, **SR**; Sporozoite Rate (Ag + Af).

**Superscripts (** ^**b**^ **) -** Total mosquitoes caught in Tano **n;** number caught.

EIRs were higher for *An. gambiae* than *An. funestus* in both impact and non-impact areas in Asutifi (Table 1). A similar trend is observed in Tano (Table 2). EIR per month in Asutifi ranged between nine infective bites/person/month and 128 ib/p/m in impact area. In the non-impact area it ranged between 6–41 ib/p/m (Table 1). EIRs in Tano ranged between 7–54 ib/p/m in impact area and 13–164 ib/p/m in non-impact area (Table 2).

### Housing characteristics and mosquito preventive measures

Between 80% - 90% of houses in the study area were roofed with corrugated zinc sheets; with 94.7% and 82.6% in impact and non-impact areas, respectively. A few houses were roofed with logs (1.8% in impact, 7.7% in non-impact) and thatch (3.5% in impact; 9.7% in non-impact) (Table 3). Over sixty percent (67.5%) of houses in impact area had no eaves gaps compared with 62.9% of houses in non-impact area. Nearly half (48%) of the houses surveyed had a bed net. In preventing mosquito bites, 22.1% of respondents used mosquito coils in impact areas as against 16.9% in non-impact areas. The use of insecticide sprays was 9.4% in impact areas and 2.8% in non-impact places. Four percent of respondents’ applied repellent creams in impact areas but a negligible 0.3% applied creams in non-impact areas. A negligible number had used strongly scented leaves (0.5%) or smoky fire (1.1%) to prevent mosquito bites in impact areas but none was recorded in non-impact areas.

**Table 3:** **Characteristics of houses and mosquito preventive measures by individuals in Ahafo area of Ghana (Nov 2006-Aug 2007)**

**Variables (N = 942)**	**Impact (N = 551)**	**Non-impact (N = 391)**	
	**n (%)**	**n (%)**	**p-value** ^**a**^
**Presence of eaves gaps**			
Yes	179 (32.5)	145 (37.1)	0.14
No	372 (67.5)	246 (62.9)	
**Type of roof**			
Corrugated zinc roof	522 (94.7)	323 (82.6)	<0.01
Mud concrete/logs roof	10 (1.8)	30 (7.7)	
Thatch roof	19 (3.5)	38 (9.7)	
**Bed net use**			
Yes	249 (45.3)	201 (51.3)	0.07
No	301 (54.7)	191 (48.7)	
**Insecticide spray**			
Yes	52 (9.4)	11 (2.8)	<0.01
No	499 (90.6)	380 (97.2)	
**Mosquito coil**			
Yes	122 (22.1)	66 (16.9)	0.05
No	429 (77.9)	325 (83.1)	
**Smoky fire**			
Yes	6 (1.1)	0 (0.0)	0.04*
No	545 (98.9)	391 (100.0)	
**Scented leaves**			
Yes	3 (0.5)	0 (0.0)	0.27*
No	548 (99.5)	391 (100.0)	
**Repellent creams**			
Yes	22 (4.0)	1 (0.3)	<0.01*
No	529 (96.0)	390 (99.7)	

*: Fisher’s Exact test.

a: Pearson Chi-squared test.

The types of construction materials correlated positively with high abundance of *Anopheles* in rooms, i.e. the presence of grass or thatch roof was associated with a significant increase in calculated odds ratio (Table 4). There was significant association in abundance of *An. gambiae* and *An. funestus* at three levels; roof type, presence of eaves gaps and bed nets in rooms of individuals. The latter association (presence of bed nets) is further explained by the fact that majority of the nets were untreated.

**Table 4:** **Factors that influence abundance of** ***Anopheles gambiae*** **and** ***Anopheles funestus*** **in rooms in Ahafo area of Ghana (Nov 2006-Aug 2007)**

**Variables**	**Number (%)**	**Odds ratio**	**95% Confidence interval**	**p**
**Presence of eaves gaps**				
No	302 (49.0)	1		
Yes	206 (63.4)	1.80	(1.37, 2.37)	<0.01
**Type of roof**				
Corrugated zinc roof	442 (52.4)	1		
Mud concrete/logs roof	25 (62.5)	1.52	(0.79, 2.92)	0.01
Thatch roof	41 (71.9)	2.33	(1.29, 4.22)	
**Bed net use**				
No	246 (50.0)	1		
Yes	262 (58.2)	1.39	(1.08, 1.80)	0.01
**Insecticide spray**				
No	468 (53.3)	1		
Yes	40 (63.5)	1.52	(0.90, 2.59)	0.12
**Mosquito coil**				
No	390 (52.1)	1		
Yes	118 (61.1)	1.44	(1.05, 1.99)	0.03
**Smoky fire**				
No	502 (53.8)	1		
Yes	6 (85.7)	5.16	(0.62, 11.05)	0.13
**Scented leaves**				
No	505 (53.8)	1		
Yes	3 (0.59)	0.44	(0.04, 4.92)	0.51
**Repellent creams**				
No	493 (53.7)	1		
Yes	15 (65.2)	1.62	(0.68, 3.85)	0.28
**Area**				
Impact	290 (52.7)	1		
Non-Impact	218 (55.6)	1.12	(0.87, 1.46)	0.38

## Discussion

The abundance of mosquitoes in the two areas (Tano and Asutifi) differed slightly. Unlike Asutifi where mosquito abundance was highest in impact area of communities, Tano experienced the reverse within the surveyed period. This observation could be attributed to micro-ecological differences and rainfall in the two areas. The total number of mosquitoes caught in this survey is high against the background of a relatively short period of collection. Similar surveys [9,10,17] in which the period of collection was longer (one or two years) using the same collection method (CDC light traps) produced lower mosquito collections.

There were more mosquitoes caught in mud/log and grass/thatched roofed houses compared to zinc corrugated roofed houses. This could be attributed to the conducive nature thatched roofed and mud houses present for mosquito entry. This observation could also be linked to the fact that these types of houses present conducive environment for mosquitoes to rest indoors. Improvement in socio-economic standards may result in improved housing architecture such as the use of window screens that can reduce malaria transmission by reducing man-vector contact. Eventually this could lead to a decreased mosquito burden inside rooms and subsequent decrease in malaria transmission levels.

EIRs in the surveyed communities demonstrate very high malaria transmission levels by *Anopheles* vectors (7–128 infective bites per person per month). This is comparable with EIRs documented in the Kintampo area where the same investigators documented 269 infective bites per person per year [18]. EIRs varied significantly between communities; therefore, control strategies need to be planned in concert with detailed communities vector indices; abundance, speciation and EIRs. In Tano, EIRs in the impact communities were significantly higher in three of seven communities while in the non-impact communities two of six communities had higher EIRs.

Since infective *An. gambiae* vectors were detected by ELISA in each month and for the entire period of the survey, it may be concluded that *An. gambiae* vectors in Asutifi and Tano are capable of sustaining an infective biting pattern throughout the year. Some communities experienced higher malaria transmission than others, which is a clear indication of how local vector populations influence malaria intensity even when the communities are not very far apart, in most cases less than 5 km apart.

*Anopheles funestus* vectors has not be incriminated as a major vector in the studied area as the numbers caught in communities were low with negligible sporozoites rates.

Mining activities is reported to have an impact on malaria vector dynamics [19]. Environmental manipulation to accommodate project-related infrastructure developments can create favourable habitats for malaria vectors, most importantly *An. gambiae* [20]*.* The Ahafo area has experienced changes since mining activities commenced and therefore environmental changes might impact on vector densities. Also, mining activities attract several categories of workers and camp followers who might create settlements with poorly constructed houses and poor drainage creating conducive grounds for malaria transmission. The changes in behaviour of reservoir hosts and the ability of pathogens to adapt to new reservoir hosts in newly-created habitats also influence the patterns of disease [21]. Integrated vector control measures [22] are appropriate even more in the context of mining areas since the environment is greatly changed by mining and human activities. Continued malaria vector monitoring is, therefore, recommended in the ongoing mining areas in Tano and Asutifi.

## Conclusions

The survey documented important malaria vector indices associated with a large mining development in a rural area. At the community level, malaria vector transmission is primarily determined by prevailing environment, housing characteristics and vector composition. Preventive measures, such as the use of treated bed nets or indoor residual spraying, need to be considered within the local context in order to maximize cost-effectiveness. For example high *kdr* + levels in key mosquito vectors will impact the design and implementation of IRS and possibly even ITN distribution. Developing a pre-project baseline is critical and helps establish the underlying community situation and facilitates a scientifically rational control programme that can be sequentially monitored. It is in both the host communities and the project’s best interest to have an objective foundation before active construction begins, during peak activity and for long-term continuous operations.
